# Navigating the transition: a multidisciplinary approach to inflammatory bowel disease in children

**DOI:** 10.1007/s00383-024-05789-8

**Published:** 2024-08-27

**Authors:** A. Raffaele, C. M. Ferlini, G. Fusi, M. V. Lenti, E. Cereda, S. M. E. Caimmi, M. Bertozzi, G. Riccipetitoni

**Affiliations:** 1 Department of Clinical, Surgical, Diagnostics and Pediatric Sciences, University of Pavia, Pavia, Italy; 2 Pediatric Surgery Unit, Department of Maternal and Child Health, Fondazione IRCCS Policlinico San Matteo, Pavia, Italy; 3 Department of Internal Medicine, Fondazione IRCCS Policlinico San Matteo, University of Pavia, Pavia, Italy; 4 Department of Internal Medicine and Medical Therapeutics, University of Pavia, Pavia, Italy; 5 Clinical Nutrition and Dietetics Unit, Department of Oncology and Hematology, Fondazione IRCCS Policlinico San Matteo, Pavia, Italy; 6 Pediatrics Unit, Department of Maternal and Child Health, Fondazione IRCCS Policlinico San Matteo, Pavia, Italy

**Keywords:** Transitional care, Inflammatory bowel disease, Ulcerative colitis, Crohn disease, Multidisciplinary approach, Dropout

## Abstract

**Purpose:**

A multidisciplinary approach to Inflammatory Bowel Disease (IBD) has recently demonstrated a positive impact in pediatric patients, reducing dropout rates and facilitating the transition to adult care. Our study aims to evaluate how this approach influences disease activity, dropout rates, and transition.

**Methods:**

We conducted a longitudinal observational study including all patients diagnosed with IBD during pediatric-adolescent age, with a minimum follow-up period of 12 months. For each patient, endpoints included therapeutic approach, need for surgery and transition features.

**Results:**

We included 19 patients: 13 with Ulcerative Colitis (UC) and 6 with Crohn’s disease (CD). Most patients required multiple lines of therapy, with over 50% in both groups receiving biological drugs. Compliance was good, with a single dropout in each group (10, 5%). The need for surgery was significantly higher in the CD group compared to the UC group (16% vs. 7.7%, p < 0.01). Mean age at transition was significantly higher in the UC group compared to the CD group (19.2 ± 0.7 years SD vs. 18.3 ± 0.6 years SD, p < 0.05).

**Conclusions:**

In our experience, the multidisciplinary approach to IBD in transition-age patients appears effective in achieving clinical remission, offering the potential to reduce therapeutic dropouts.

## Introduction

The transition from pediatric to adult care is defined as a "planned and intentional movement of adolescents and young adults with chronic conditions from child-centered healthcare systems to adult-centered healthcare systems" [1]; the goals of the transition are to "maximize independence and promote self-determination" [2]. Pediatric care models are typically family-centered, with particular attention given to growth, puberty, school, and psychosocial needs [3]. In contrast, adult patient medical care is more often centered on the patient and on a specific condition, and the scope for addressing work-related, social, and emotional issues is more limited [4–6]. In this context the patient's autonomy is required, both in making decisions and in adhering to the therapy according to the doctor’s instructions. The transition process therefore requires a multidisciplinary approach; tools for assessing the transition focus primarily on disease knowledge [7], but also on self-efficacy and resilience.

Based on these premises, a multidisciplinary IBD Transition Clinic was established in April 2021 at the Pediatric Clinic of the I.R.C.C.S. Policlinico San Matteo Foundation in Pavia. The clinic involves collaboration among a pediatrician, a gastroenterologist, a pediatric surgeon, and a nutritionist. Patients have been followed until the age of 18–20, at which point transition to adult gastroenterology clinics was implemented.

The aim of this study is to analyze our experience navigating how the multidisciplinary approach influences transition to adult care in Crohn's Disease and Ulcerative Colitis, with a focus on characterizing the peculiarities of IBDs in adolescent subjects. Our goal is to optimize patient care and outcomes, thus addressing an unmet need in the existing medical literature.

## Methods

We performed a single-center, longitudinal observational study. All patients diagnosed with IBD during pediatric age and attending our multidisciplinary clinic were included. Patients were evaluated at disease onset and at the last follow-up in March 2024.

Inclusion criteria for the study were: confirmed diagnosis of IBD, attendance at the multidisciplinary IBD Transition Clinic with at least 1 year of complete follow-up, signed informed consent. Exclusion criteria were: inconclusive or uncertain diagnosis of IBD, patients not attending the multidisciplinary IBD Transition Clinic, refusal to participate in the study.

The descriptive variables analyzed were: gender, type of IBD, age at onset, BMI at onset, family history of IBD, family history of autoimmune disease, disease duration, clinical manifestations at onset (abdominal pain, abdominal distension, abdominal mass, diarrhea, hematochezia, nausea, vomiting, weight loss, anorexia, fever, arthralgia, ankylosing spondylitis, sacroiliitis, erythema nodosum, pyoderma gangrenosum, conjunctivitis, uveitis, episcleritis), localization and phenotypic behavior of CD, extent and severity of UC, therapy lines, need for surgical treatment, baseline and follow-up C-reactive protein (CRP), baseline and follow-up fecal calprotectin.

For patients who reach adulthood during the observation period, we evaluated actual transitional passage to the adult care setting and therapeutic drop-out.

## Results

We included 19 patients: 13 with UC (68.4%) and 6 with CD (31.6%). The need for surgery was significantly higher in CD vs UC group: 33% vs 7.7%, p < 0.01. Mean age at transition was significantly higher in UC group vs Crohn group: 19.2 ± 0.7 years SD vs 18.3 ± 0.6 years SD, p < 0.05. Dropout rate was similar in both group, with a cumulative dropout rate was 10.5%. Below we illustrate some peculiar aspects of each group.

### Ulcerative colitis (UC)

We treated a total of 13 patients, 7 female (53.8%) and 6 male (46.2%), average age at onset 11.9 ± 2.4 years SD, average BMI at onset 17.8 ± 1.27 kg SD. One patient had familiarity for autoimmune disease (AD)/IBD (7.7%). Main symptoms at onset were rectorrhage (92%), diarrhoea (46%), abdominal pain (23%) and asthenia (15%). The majority of patients required multiple lines of therapy: mesalazine 50%, biological drugs 53%, steroids 46% and immunosuppressive drugs 46%. Overall compliance to therapy was good. One patient required surgical intervention (7.7%). Analysis of changes in laboratory markes showed a reduction of CRP (p 0.06) and Calprotectin (p 0.08). At follow up, 4 patients (30%) completed the transition towards the adult clinic, at a mean age of 19.2 ± 0.7 years SD and 1 dropout (7.7%) was registered.

### Crohn disease (CD)

In this group there is majority of male (5 patients, 83.4%) and only 1 female (16.6%), average age at onset 13.1 ± 3 years SD, average BMI at onset 17 ± 2.6 kg SD. One patient had familiarity for AD/IBD (16.6%). Main symptoms at onset were diarrhoea (66%), recurrent abdominal pain (66%), perineal disease (50%), loss of weight (33%) and rectorrhage (16%). The majority of patients required multiple lines of therapy: biological drugs 66%, mesalazine 50%, immunosuppressive drugs 33%, and steroids 16%. Compliance to therapy was good. Two patient required surgical intervention (33%). Analysis of changes in laboratory markes showed a reduction of CRP (p < 0.05) and Calprotectine (p 0.08). At follow up, 4 patients (66%) completed the transition towards the adult clinic, at a mean age of 18.3 ± 0.6 years SD and 1 dropout (16%) was registered.

## Discussion

The presence of a multidisciplinary care setting, in addition to facilitating the transition process, allows for the analysis of the peculiarities of pediatric and transitional IBDs, with a focus on disease manifestations and clinical and therapeutic outcomes. Furthermore, it has enabled the evaluation of each patient's ability to transition to an adult care setting. Despite the limited size of our sample, given the young age of our clinic, interesting data and valuable discussion points have emerged from this initial analysis.

In our population, UC was predominant (68.4%), with a low percentage of the patients presenting family history for IBD, contrary to past evidence reported in scientific literature. However, the epidemiological distribution confirmed a male prevalence for CD (M:F = 5:1), and an unclear sex prevalence for UC (M:F = 1:1) in the sample age group. IBDs presented at a mean age of 12.3 years and in patients with a mean BMI of 17.6 kg/m2, indicating patients were mildly underweight. The most evident disease manifestations were abdominal pain, diarrhea, and hematochezia, which is consistent with available literature [8–10].

Considering the severe disease phenotype, most of our sample underwent multiple lines of therapy, despite their young age. However, biological drugs were often suspended due to side effects or poor response, and needed optimization. Poor response to monoclonal antibodies in pediatric patients seems correlated with a severe disease phenotype, stricturing CD, prior intestinal resections, and disease duration exceeding 2 years, conditions characterizing some of our patients [11–14].

Additionally, the majority of patients showed a good compliance to therapy, with only 3 patients (15%) that were non-adherent to therapy, especially near the transition age, possibly due to medication side effects, perceived lack of therapeutic effect, subjective well-being, or simply forgetfulness. Moreover, in the case of 2 CD patients, parents were the ones who refused to accept the proposed therapy despite the patients being at least somewhat willing to continue treatment; in one case parental opposition was eventually a decisive factor in causing dropout.

Disease activity assessment included blood and fecal examinations, showing a reduction in mean values of CRP and fecal calprotectin between disease onset and the last follow-up. Only the difference in mean CRP values in CD group has resulted statistically significant (p < 0.05). Despite disease severity and the need for multiple therapeutic lines, clinical and laboratory markers suggest good overall IBD control.

Regarding transition to adult care, the strength of the multidisciplinary team is undeniable, as other Authors reports [3, 15–18]. In our sample, young adults were not abruptly shifted to a new clinic with adult specialists, devoid of parental support. By benefiting from multiple specialists for several months, both patients and families were guided step by step through the disease acceptance and management process. They also began to familiarize themselves with adult specialists, particularly gastroenterologists and nutritionists, who would later take over their care. The group collectively evaluated each individual's adequate preparation for transition, resulting in eight successful transitions to date. For other individuals, the multidisciplinary assessment allowed for timely identification of potential red flags, such as inability to self-regulate, risking therapy suspension without parental involvement, or excessive parental attachment, necessitating additional psychological support. We believe that this careful assessment and continuity of the multidisciplinary pathway have significantly influenced the prevention of possible therapeutic dropouts, whose rates are reported to be as high as 30% even in studies involving a transitional program versus 10.5% in our IBD sample [19].

Of note, patient in the UC group showed a higher mean age at transition than those in the CD group, and the difference was statistically significant (p = 0.02). The complexity and number of treatments right from the start, the need to perceive a “familial” and caring therapeutic environment and the possibility of undergoing invasive diagnostic tests under sedation due to the paediatric setting (which might not be granted in adult care) may all be elements that could encourage patients to delay transition. However no readily apparent explanation suggests why this happened on average later in our UC sample, which begs the need for further inquiry.

## Limitations and strengths

While this study has limitations, it also holds great potential. The main limitation is the sample size, although compatible with a recently established clinic and the incidence of IBDs in the analyzed age group. Additionally, a comparison of endoscopic evaluations at onset and follow-up would have provided further objective data on disease activity. However, frequent endoscopic examinations in pediatric patients are challenging due to their invasiveness and the need for anesthesia. Since clinical response guides the need for endoscopic reevaluation and not all included patients had follow-up exams available, this data was not evaluated at this time. Nevertheless, we believe that multidisciplinary activity can still provide adequate information over time. According to our multidisciplinary experience with other pathologies, we are strongly convinced that standardized protocols for transitional care in specialized centers, involving both paediatric surgeons and adult care providers, will be able to improve awareness about these patients’ needs [20]

## Conclusions

In transitional age, IBDs pose a challenge for physicians. This age group is delicate in terms of family dynamics, emotional, relational, and psychological aspects. The clinical presentation of the disease itself has unique characteristics compared to onset in adulthood, and therapy adherence is still heavily influenced by caregivers. Therefore, the physician's task is to gradually empower patients regarding their illness and the necessary chronic care.

Our study has highlighted the initial results of a multidisciplinary clinic initiated based on increasing scientific evidence supporting the benefits of a multidisciplinary approach to IBD in transitional-age individuals. This approach aims to guide young patients gradually towards adult care. Despite the need for multiple lines of therapy, clinical remission data, and hence therapeutic response, are encouraging. Thus, continued follow-up is necessary to investigate the long-term course of the disease.

## Data Availability

The data supporting the findings of this study are available from IRCCS Policlinico San Matteo archive. Due to privacy concerns, patient data are available in a de-identified form. For further inquiries, please contact Corresponding Author.
